# mSphere of Influence: Learning Biology from the Radio

**DOI:** 10.1128/msphere.00026-23

**Published:** 2023-02-02

**Authors:** Daniel Schultz

**Affiliations:** a Department of Microbiology and Immunology, Dartmouth - Geisel School of Medicine, Hanover, New Hampshire, USA

**Keywords:** bioengineering, cell biology, circuit design

## Abstract

Daniel Schultz works at the intersection of microbiology and biological physics. In this mSphere of Influence article, he reflects on how two papers concerning the radio, “Can a biologist fix a radio? Or, what I learned while studying apoptosis” by Yuri Lazebnik and “The evolved radio and its implications for modeling the evolution of novel sensors” by Jon Bird and Paul Layzell, offered him complementary perspectives on how to bridge the gap between engineering and biology in the study of cellular circuits.

## COMMENTARY

Having started my career as an electrical engineer, I decided to write about the radio and how it helps us think about microbiology. If you look inside a radio, you will find in those green circuit boards an impenetrable mess of different components, connected in mysterious ways to perform complex functions, much like what you will find inside a living cell. The radio is basically a signal transduction pathway, receiving electromagnetic waves and converting them into sound. Unlike the cell, however, a radio comes with an instruction manual. Those circuits are not the result of a chaotic evolutionary process but were purposefully designed and built by engineers, reflecting our best knowledge of electronics. Since we’d like to achieve such mastery of biological circuits someday, this is a useful analogy to think about our approaches to studying cell biology. Would biological approaches work on the radio? Can we use engineering approaches to study the cell? Here, I comment on two papers, one by a biologist and one by engineers, that offer complementary perspectives on these questions.

In a humorous critic of the traditional paradigms of biological research (1), Yuri Lazebnik asks, “Can a biologist fix a broken radio?” With no prior knowledge of electronics, he says, biologists would start by categorizing all of the different components in a healthy wild-type radio by shape, size, and color. Next, they would try to remove one component at a time, assigning specific phenotypes to all components that would cause the radio to malfunction. In a lucky break, they would find an essential component without which the radio does not function, and further studies would show that it connects the rest of the radio to a long extendable metal object. They would then find that the longer that object the better, which would provide an evolutionary explanation for why it is extendable. And so the experiments would proceed until all components are catalogued, all connections are described, and the consequences of removing each component are documented.

But in the end, would the biologist be able to fix the radio? Maybe. If a known component is clearly broken, the biologist could try his luck by replacing the faulty component with a similar one. However, if the components have tunable properties and the circuit is misadjusted, then the radio cannot be fixed without a deeper understanding of how the different components work together to control the flow of information. As the author puts it, a problem that requires calculus cannot be solved with just arithmetic and another set of experiments (1). Therefore, it is certainly worthwhile to try to extend our highly successful quantitative descriptions of electronic circuits to biological ones. This is not a new idea, and much progress has been made. But are biological systems organized in the same way? Or are they too complex and special to be tamed by such descriptions?

Addressing these questions, Jon Bird and Paul Layzell report their efforts in incorporating evolution into the design of electronic circuits (2). Engineers have long used “evolutionary algorithms” to solve some classes of optimization problems. Such algorithms use mechanisms inspired by biological evolution, such as reproduction, mutation, recombination, and selection, to numerically optimize parameters in systems with large sets of constraints. This time, however, the authors went beyond computer algorithms, constructing physical hardware capable of evolving by creating and changing connections between electronic components while evaluating function according to some metric. Different from a simulation, in which circuits evolve with an idealized environment and constraints created by the programmer, such physical circuits are free to take advantage of subtle properties of the real world.

To test their device, the authors decided to start by evolving something familiar, and opted for an ascillator with a precise frequency. To prevent trivial solutions, the circuit was not allowed to utilize capacitors, which are typically used to define time constants in electronic circuits. Therefore, oscillations were expected to result from unlikely recurrent loops of digital gates or operational amplifiers. The success of the evolutionary process was very surprising, with one-half of the runs attaining the target frequency within 1% and with good amplitude. However, the evolved architectures did not resemble any circuits known to produce oscillations. The expected loops were nowhere to be found. Intriguingly, simulations of the evolved circuits failed to oscillate, even after incorporation of all of the parasitic capacitance expected to exist within the physical components. The circuits were apparently sensitive to even tiny transients in their voltage supply and stopped working when the oscilloscope used to measure their behavior, or even a nearby soldering iron, was unplugged from the wall.

After much scratching of their heads, the authors realized what had happened. Instead of evolving an oscillator, they had evolved a radio! The circuit was picking up oscillations from a nearby computer, using a long printed track in the circuit board as an antenna and the oscilloscope as ground voltage. This experiment shows how real-world evolution can think outside our narrow-minded boxes, exploring novel designs in ways that are difficult to anticipate. Paraphrasing the authors, evolution will potentially exploit any physical characteristic that can influence circuit behavior, and these characteristics are present in the entire evolutionary environment. I note here that, when the engineers failed to understand the evolved electronic circuits using their traditional tools, they resorted to methods much like the caricature biologist described above, unplugging random components in hopes of gaining any valuable insights. But then, to their credit, they did quickly find all the parts of a radio when they finally recognized one.

Therefore, biological circuits are special after all. While engineered circuits are neatly organized in functional modules, biological systems are often interconnected and cross talk in nonobvious ways. They are also noisy, plastic, parallelized, they span different scales, and their function is not always well defined. Our notions of circuit analysis and design from electronics are a good start, but biology will require new language. Having migrated from physics to microbiology, a large part of my work is to find this common language. Although the line between these communities continues to be blurred over time, there is still a wide gap to breach. I felt that for the first time while still in grad school, while failing miserably at trying to excite a biologist friend about our analytical solution for the stochastic description of a self-regulating gene (3) (still one of my most cited papers). “What is that good for?” At the time, she found all my answers very unsatisfying. I like to think the day is coming when this utility will be more obvious (and maybe my argumentation more compelling). We’re getting there.
